# Transition-state theory predicts clogging at the microscale

**DOI:** 10.1038/srep28450

**Published:** 2016-06-22

**Authors:** T. van de Laar, S. ten Klooster, K. Schroën, J. Sprakel

**Affiliations:** 1 Physical Chemistry and Soft Matter, Wageningen University, Wageningen, The Netherlands; 2 Laboratory of Food Process Engineering, Wageningen University, Wageningen, The Netherlands

## Abstract

Clogging is one of the main failure mechanisms encountered in industrial processes such as membrane filtration. Our understanding of the factors that govern the build-up of fouling layers and the emergence of clogs is largely incomplete, so that prevention of clogging remains an immense and costly challenge. In this paper we use a microfluidic model combined with quantitative real-time imaging to explore the influence of pore geometry and particle interactions on suspension clogging in constrictions, two crucial factors which remain relatively unexplored. We find a distinct dependence of the clogging rate on the entrance angle to a membrane pore which we explain quantitatively by deriving a model, based on transition-state theory, which describes the effect of viscous forces on the rate with which particles accumulate at the channel walls. With the same model we can also predict the effect of the particle interaction potential on the clogging rate. In both cases we find excellent agreement between our experimental data and theory. A better understanding of these clogging mechanisms and the influence of design parameters could form a stepping stone to delay or prevent clogging by rational membrane design.

Clogging is encountered at many length scales, ranging from the deposition of marginally-soluble asphaltenes at pipe walls in oil recovery^1^, the formation of protein fouling layers in waste water treatment^2^,^3^, particle clogging during membrane filtration^4^ or microfluidic operations^5^,^6^,^7^. Similar phenomena are encountered at much larger length scales such as in blockades of granular hopper flows^8^, the emergence of traffic jams on merging lanes^9^,^10^ or in crowds swarming through narrow escape routes^11^,^12^. It is speculated that the same physical principles govern the obstruction of flow through a narrow passage in many of these scenarios irrespective of their scale^13^. In all of these cases, preventing clogging is an immense challenge due to its often severe, costly and energy-consuming consequences. Yet this remains difficult as the generic mechanisms with which permeating flows become hindered or blocked remain largely unknown. This is especially the case for clogging at the microscopic scale as encountered during a plethora of membrane filtration processes^4^.

At the microscale, clogging typically results from the accumulation of molecules or dispersed particles at a membrane surface, leading to the build-up of fouling layers; initially these reduce the permeability of channels or pores and ultimately lead to a complete blockage of the flow^4^. Fouling and clogging form one of the major sources of efficiency loss in membrane filtration processes. Remediating the formation of fouling layers and clogging as a whole currently requires complete cessation of the process, and the use of energy- and time-consuming cleaning strategies before the operation can be resumed^14^. Since the propensity of a certain flow geometry to clog depends on the ratio of its characteristic dimension to that of the particles or molecules which accumulate over time, clogging is particularly severe in microstructured devices; in addition to the obvious importance for membrane processes, the rise of microfluidics as an emerging technology makes it increasingly urgent to resolve^15^.

To enable the development of effective strategies to delay fouling and the clogging that ensues, or to prevent it from occurring altogether, a deep understanding of the fundamental mechanisms that leads to this major source of efficiency-loss is essential, yet very incomplete to date. The size ratio of particles and constriction plays an important role, where two extremes can be identified. Either a single particle can block a constriction, for instance larger contaminants in a suspension of smaller particles^16^ where these contaminants almost completely determine the rate of clogging. Or the case in which multiple particles are required to form an agglomerate large enough to cause a clog, so that the actual ratio between particle size and constriction width strongly determines the time it takes for clogging to occur. This results in a clogging process that appears to depend solely on the number of particles that pass through a constriction^6^. However, little is known about the influence of particle-particle and particle-wall interactions^17^ and the geometry of the constrictions themselves^13^.

In this paper we explore the influence of pore design and particle interactions on the clogging rate in dilute suspensions. We study clog formation experimentally using multiplexed microfluidic models for dead-end filtration and quantitative imaging. We observe a strong dependency of the clogging rate on both geometry and attraction strength. To account for these effects we derive an analytical model based on transition-state theory which provides a quantitative and predictive description of our experimental data.

## Results and Discussion

**Figure 1:**
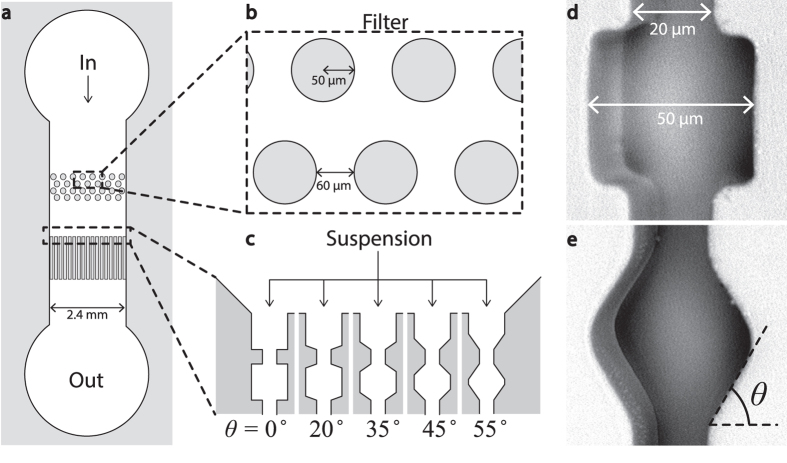
Overview of the multiplexed dead-end filtration micromodel: (**a**) an overview of the device geometry, (**b**) design of a pre-device filter which captures large contaminants such as dust or PDMS debris from the fluid, (**c**) a schematic illustration of the five different entrance angles studied. Each channel features a series of 19 constrictions with identical geometry along the flow direction. For statistics each channel type is repeated 6 times on each device; this allows 6-repeat measurements and 5 different geometries to be explored in a single experiment. (**d**) Scanning Electron Microscopy (SEM) picture of a *θ* = 0° constriction with its dimensions indicated and (**e**) a SEM picture of a *θ* = 55° constriction with the entrance pore angle *θ* defined as indicated.

We study clogging using a microfluidic device as a filtration micromodel, inspired by previous studies^5^,^6^,^7^,^16^. These filtration micromodels mimics dead-end filtration. Our device consists of thirty channels in parallel; each channel consists of 19 constrictions in series with a width of 20 *μm*. These constrictions simulate the membrane pores and are the main site of clogging events. The thirty channels, situated next to each other, are divided in five sections of six channels. We vary the entrance pore angles, defined as *θ*, of the constrictions perpendicular to the flow direction between the different sections with *θ* = 55°, 45°, 35°, 20° and 0°. The distance between the constrictions in series (along the flow direction) is kept constant at 50 *μm*. We add a contaminant filter a few hundred micrometer upstream from the entrance of the device to remove large contaminants, such as dust, as these are reported to have a strong effect on the experimental observations^16^. An overview of the entire device is shown in Fig. 1.

**Figure 2:**
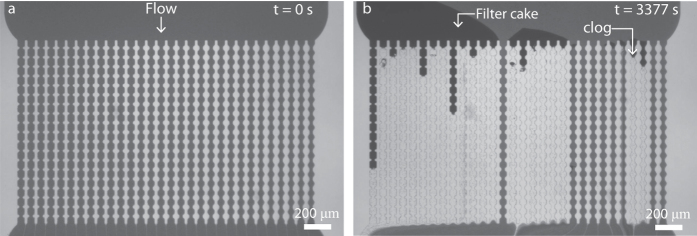
Brightfield microscopy images at t = 0 (**a**) and t = 3377 s (**b**) for a repulsive suspensions, in which flow direction, filter cake and a clog are indicated with arrows. Grey channels are perfused with the suspension; as a clog appears, the channel becomes white down-stream, as particle flow is blocked, while the channel turns dark upstream from the clog due to the accumulation of excess particles.

Upon flowing a purely repulsive suspension of particles, with a diameter of approximately 1/10 of the pore diameter, through our filtration micromodel, we observe a distinct sequence of events. Initially, all channels are permeated by the suspension. After some time, clogs begin to appear, after which that channel, upstream from the clog, becomes filled with a layer of densely packed particles, whereas only solvent permeates downstream from the blockade. This can be easily seen in our experimental images (Fig. 2). As the flow continues, a filter cake forms due to the accumulation of particles towards the entrance of the filtration micromodel.

To characterise the statistics of clogging, we use automated image analysis to identify which channels clog when. To improve statistics, we repeat these measurements with at least 3 identical devices. We can now plot the fraction of channels *α* which has clogged as a function of time. For the purely repulsive case, we see how *α* grows steadily over the course of several hours. These timescales are considerably larger than those reported previously for a similar geometry with *θ* = 0° 6, which we attribute to the addition of a surfactant in our experiments. This enhances the colloidal stability and prevents particle absorption to the PDMS walls^17^.

As the formation of a clog requires the accumulation of multiple particles at the clogging site, a minimum clogging time must exist. In other words, at least the number of particles required to form a pore-spanning aggregate must have passed for clogging to occur. In our experiments, we flow the suspensions at high fluxes, such that this minimum time is very short compared to our experimental resolution; as such, we do not observe it with statistical significance here.

To quantify these data, we fit the experimental results to a Weibull model, often used to describe the kinetics of failure processes^18^:


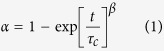


in which, *τ*_*c*_ is the characteristic clogging time and *β* the stretch exponent, which reveals information about the underlying failure lifetime distribution. We find that *β* is close to unity for all values of *θ*; this indicates a well defined failure lifetime with a narrow distribution.

**Figure 3:**
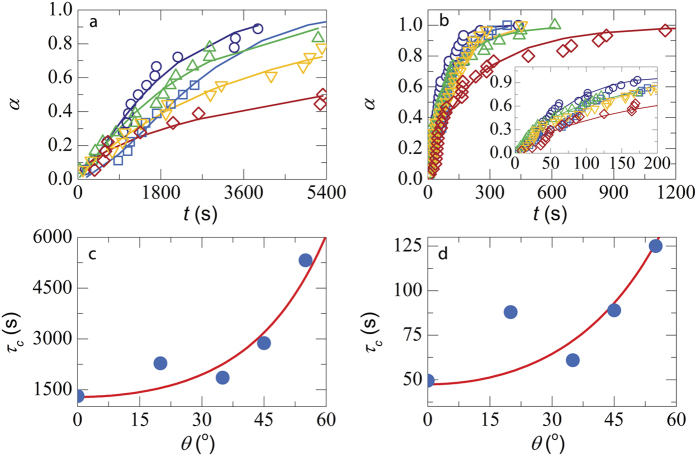
(**a,b**) Fraction of clogged channels (*α*) as function of time *t* for a repulsive system (**a**) and one in which depletion attraction has been induced with *U*_*dep*_ = 4.0 *k*_*B*_*T* (**b**) for different entrance angles *θ* = 0° (purple circle), 20° (blue square), 35° (green triangle), 45° (yellow triangle) and 55° (red diamond); lines are fits to a Weibull model. (**c,d**) Characteristic clogging time *τ*_*c*_ (symbols) as function of *θ* for the repulsive (**c**) and attractive system (**d**). Drawn lines are a fit to the transition-state model of Equation 7.

We find a clear impact of the pore geometry on the rate of failure of our micromodel. As the entrance angle becomes steeper, *θ* → 0, the characteristic clogging time decreases by almost a factor 4 as compared to the most shallow angle we study at *θ* = 55° (Fig. 3c). A similar trend was observed previously for athermal grains passing through a hopper orifice^19^.

**Figure 4:**
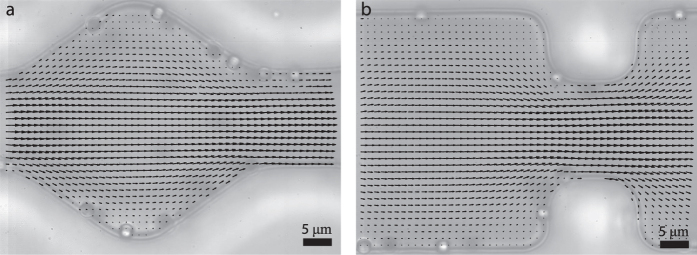
Flow profiles, as determined by PIV, of a shallow (**a**, *θ* = 55°) and sharp entrance angle (**b**, *θ* = 0°).

The effect of channel shape on the local flow field may be responsible for the strong dependence of the failure rate on pore geometry. Our experiments are conducted at fluid Reynolds numbers around ~0.4; it is thus possible that corner vortices, or stagnant pockets develop for steeper entrance angles. To explore this hypothesis, we determine the flow field around a single pore entrance using particle imaging velocimetry (PIV). Interestingly, we see no deviations from laminar flow, within our experimental resolution of ~1 *μ*m, both for shallow and steep entrance angles (Fig. 4). This illustrates, how local alterations of the flow field cannot explain the observed angular dependency.

**Figure 5:**
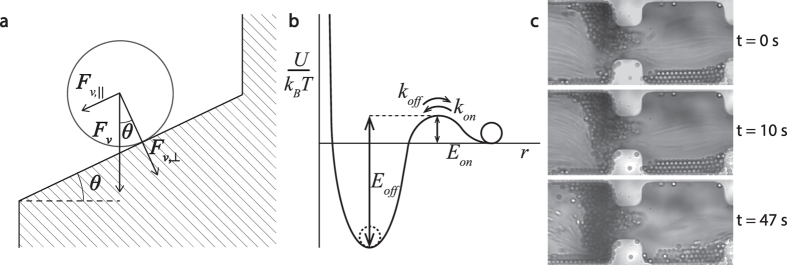
Schematic illustration of the angle-dependent viscous forces acting on a particle close to the wall (**a**) and the particle interaction potential with *k*_*on*_ and *k*_*off*_ the natural rate of agglomeration and detachment and *E*_*on*_ and *E*_*off*_ the corresponding energy barriers. (**c**) a zoomed-in, time series of a clog forming at a single constriction, with flow from left to right.

Rather, we realise that clogging in the limit we investigate, i.e. where the particle size is smaller than the pore diameter, must be accompanied by the formation of particle bridges spanning the width of the channel. In turn, this must be the result of particle-wall and subsequent particle-particle aggregation. We note that at rest, these colloidal suspensions do not show any signs of particle aggregation; thus, the observed effects result purely from the combined action of confinement and flow. At the low volume fractions we study, the emergence of configurational arches, often found for granular flows^19^, can be ruled out. The potential energy of interaction between a colloidal particle and the solid wall, or between two particles, is composed of two opposing terms; at short distances, the particles will experience an attractive force due to van der Waals interactions. Typically, these van der Waals interactions for micronsized particles are sufficiently strong to induce irreversible aggregation. However, the surfactant adsorbed onto the particle surface, provides a steric repulsion that keeps the particle stabilized for some time. The combination of these two terms, results in a characteristic energy barrier of height *E*_*on*_, that needs to be crossed before aggregation can occur (see Fig. 5b).

As a result, aggregation is thermally-activated and occurs at a rate that can be described by an Eyring-type equation as:


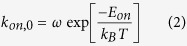


in which *ω* is the attempt frequency, which can be related to the characteristic frequency of Brownian motion. In analogy, the spontaneous detachment of a particle from the wall or from a particle-particle pair can be described as


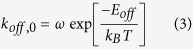


in which *E*_*off*_ is the energy barrier for particle dissociation.

Under flow, particles also experience a viscous force due to fluid flow around their impermeable surface. This can be approximated using the Stokes drag, neglecting lubrication effects, as *F*_*v*_ = 6*πηav*, in which *η* is the fluid viscosity, *a* the particle radius and *v* the flow velocity of the fluid relative to the particle. For a particle which resides at a wall that is inclined at an angle *θ* with respect to the pore entrance, the viscous force can be decomposed in a contribution perpendicular to the surface, *F*_*v*,⊥_ = *F*_*v*_cos*θ*, which pushes the particle against the wall, and a shearing-force which enhances detachment that acts parallel to the surface: *F*_*v*,||_ = *F*_*v*_sin*θ*, as illustrated in Fig. 5a. For small particles, the particle Reynolds number is low (in our experiments *Re*_*p*_ ~ 6 ⋅ 10^−3^), such that inertial lift can be ignored^20^.

These hydrodynamic forces acting on particles at the channel walls will alter the agglomeration kinetics. To describe this, we adopt the transition-state approach of Kramers^21^,^22^:


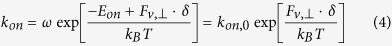


where *δ* is the activation length, which is the range of the attractive Van Der Waals interactions, typically of order of one to a few nanometers. In an similar way, the hydrodynamic forces on the particles that act parallel to the wall, and thus perpendicular to the wall-particle bond, will aid in the natural rate of particle dissociation:


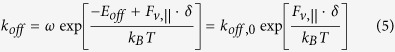


in which *E*_*off*_ is the depth of the attractive Van Der Waals minimum. The balance between particle attachment and dissociation gives a total rate of particle accumulation as:


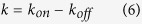


For the experiments we describe here, we use relatively large polystyrene particles, whose Van Der Waals interactions are very strong *E*_*off*_ ≫ *k*_*B*_*T*. As a result *k*_*off*_ ≈ 0, so *k* ≈ *k*_*on*_ and therefore agglomeration will be irreversible. For a clog to appear, a sequence of several particle aggregation events must occur; this is also what we observe experimentally in a close-up of a forming clog at a single constriction (Fig. 5c). Single aggregation events occur at a rate *k*; assuming that subsequent events are independent, the scaling of the overall clogging time is expected to follow *τ*_*c*_ ∝ 1/*k*. This leads to the following prediction for the characteristic clogging time:


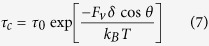


in which *τ*_0_ ∝ 1/*k*_*on*,0_ is the characteristic time for particle agglomeration in absence of flow enhancement. We find an excellent agreement between our theoretical prediction and the experimental data (line Fig. 3c). In this case, for surfactant stabilised polystyrene particles we find a *τ*_0_ = 2.9 * 10^4^ s, reflecting the stability of the suspension towards spontaneous aggregation, and a total work performed by the drag forces to enhance agglomeration of *F*_*v*_*δ* = 3.2 *k*_*B*_*T*.

It has been shown previously^6^,^13^ that reducing the repulsive barrier between particles strongly increases the propensity for clogging. Interestingly, our equation allows us to predict these effects by means of the effective energy barrier that particles need to cross before aggregating. Introducing an additional attractive force between the particles should reduce the energy barrier and thus enhance clogging. To explore this, we introduce a depletion interaction between the particles of *U*_*dep*_ ~ 4.0 *k*_*B*_*T*; we predict that this affects the quiescent agglomeration time as 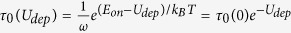 = 470 s.

For the attractive system, we perform the same experiments and data analysis and indeed see a strong reduction in the failure time of the micromodel (Fig. 3b). While the characteristic clogging time is two orders of magnitude lower, it shows the same dependency on entrance geometry. Interestingly, without additional fitting parameters our theory now quantitatively fits the experimental data (Fig. 3d). Please note that we observe a distinct outlier in our data at a pore angle *θ* = 20°; further investigation is required to evaluate if this effect is significant and what could be at its origin. Nonetheless, these data illustrate clearly how clogging can be strongly delayed by designing a membrane or filtration device with the appropriate geometry.

**Figure 6:**
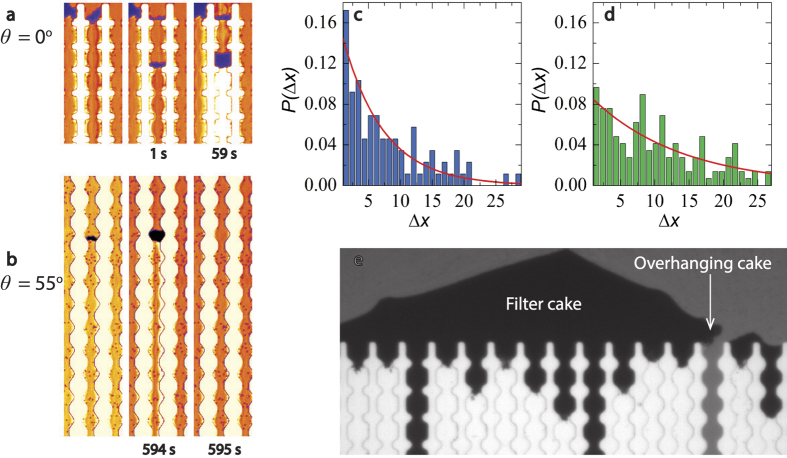
Experimental data showing detachment and displacement of an entire clog either to a new position downstream (**a**) or leading to complete declogging of the channel (**b**). Probability of the next clogging event occuring a distance Δ*x* away, with *x* in units of channel number, for the repulsive (**c**) and attractive systems (**d**). Close-up brightfield microscopy image of a filtercake with overhang at the verge of blocking the neighboring channel (**e**).

So far, we consider clogging as a process in which an independent sequence of events leads to the blockade of flow. While this allows us to make quantitative predictions of the clogging rates, we observe some cooperative effects in our experiments. First of all, we find cases where clogging of a particular channel exhibits intermittency. A clog which is formed sometimes detaches from the device walls in its entirety (Fig. 6a,b). For the shallow angles, this occasionally leads to a full reestablishing of the permeating flow through that channel. For the steeper angles however, the clog only travels a few pores down where it anchors again.

Once a pore gets blocked, the permeation of fluid continues whereas particles cannot permeate further. As a result, a distinct filter cake develops upstream from a clog. If the filter cake grows until the top of the channel, it can form an overhanging structure, which in turn could affect the probability for the neighboring channels to clog (Fig. 6e). To investigate this quantitatively, we determine the probability *P*(Δ*x*) that the next clogging event occurs at a distance Δ*x*, where *x* is the channel number. If clogging of a single channel is fully independent, we should expect *P* to be independent of Δ*x*. By contrast, we find a strong exponential dependence of the clog probability; this implies that it is more likely that neighboring channels clog in sequence, somewhat like a nucleation-and-growth scenario, rather than this happening as individual and uncorrelated events. It is important to note however, that this effect is subtle as compared to the large effect of pore geometry; thus, the pore geometry dependence cannot be explained only by this cooperative effect but must result from the viscous force-induced particle agglomeration as discussed above. Finally, we observe that the cooperative effect of the filter cake is much stronger for the repulsive case, which clogs much slower. This can be understood by considering the timescale of clogging relative to the time it takes to build up a filter cake. In dead-end filtration, the latter is governed only by the fluid flux and particle concentration, and is thus independent of attraction strength. If clogging occurs rapidly as compared to the build-up of the cake, its effect will be small (Fig. 6d). If clogging is slow, the cake has ample time to form and will have a stronger effect on the spatial correlation of clogging events (Fig. 6c).

## Conclusion

In this paper we describe how the mechanisms that underly particle clogging in dead-end filtration can be experimentally unravelled and quantitatively modelled. We show how, even though cooperative effects are observable, the characteristic failure rate of a filtration membrane can be understood based on the theory agglomeration kinetics of individual particles. Not only, does this allow prediction of the strong geometry dependence of clog formation, but also of the effect of interparticle interactions. While the micromodel presented here represents a highly idealised picture of a dead-end filtration membrane, several extensions could be imagined for future research to include complexity that is found in realistic membrane systems. For example, wall roughness, polydispersity in pore sizes or distance between pores, must be expected to have significant effects on the rate and spatial correlations of clogging. In principle, these effects could be explored with relative ease in the approach we described here. Moreover, with the microfluidic approach we use, introducing a crossflowing fluid across the membrane surface is feasible; since such a crossflow will in particular effect the filter cake build-up, it is expected to have strong effects on the cooperativity in clog events we discussed. Extending this approach, combining experimental observation on well-defined model systems and analytical theory, could lead to a deeper understanding of membrane failure and in the future provide new design rules for novel membrane systems with improved operational lifetime.

## Materials and Methods

### Suspensions

We use a 4 wt% suspension of monodisperse polystyrene particles with a diameter of 3 *μm* in a density-matching mixture of 45 vol% water and 55 vol% heavy water. We add 0.1 wt% pluronic F127 as a surfactant to sterically stabilise the particles. We synthesize the polystyrene particles by dispersion polymerisation^23^. In 150 ml butanol we dissolve 17 ml styrene monomer, 2.34 g poly(vinylpyrrolidinone)-k30, 0.64 g dioctyl sulfosuccinate sodium salt (AOT) and 0.170 g 2, 2-azobis(2-methylpropionitrile) (AIBN). After mixing we purge the solution with nitrogen for 20 minutes and subsequently evacuate the round bottom flask. We allow the reaction to proceed overnight at 70 °C. The particles are cleaned by repeated centrifugation and resuspension.

Polystyrene is well suited for this particular experiment due to the large mismatch in refractive index with the aqueous medium providing strong light scattering which we use for automated image analysis of clogging events. The particle size and concentration are chosen such that experimental clogging times are not excessively long, based on the data in^6^.

In addition to the repulsive system, we also study clogging upon inducing an additional attractive force between our sterically-stabilised particles. We do so by means of the depletion interaction, that arises when small non-adsorbing colloidal particles, in this case silica nanoparticles (Ludox TM-40), are introduced to a suspension of larger microparticles^24^. The resulting attraction strength can be calculated as^25^:


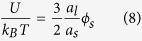


where 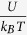 is the attraction strength in units of the thermal energy *k*_*B*_*T*, *a*_*l*_ and *a*_*s*_ are the radii of the larger polystyrene particles and smaller Ludox particles respectively (*a*_*s*_ ~ 7 *nm*) and *ϕ*_*s*_ is the volume fraction of the Ludox particles. For our experiments we choose an attraction strength *U* ~ 4*k*_*B*_*T*.

### Microfluidic experiments

We fabricate microfluidic devices following standard soft lithography methods^26^ replica-templated from Sylgard 184 silicone rubber at a mixing ratio of 10:1. The PDMS is cured at 65° for at least 1.5 hours. We bond the PDMS devices onto glass microscopy slides following plasma treatment. After connecting the tubing, we flush the device with ethanol and water to remove any large contiminants which remain after device preparation. In all cases, the height of the devices is 40 *μ*m. We flow the suspension through our devices with a constant pressure of 100 mbar, controlled with an accuracy of 0.1 mbar, applied by an Elveflow OB1-MK3. By working at a constant pressure drop across the device the flow velocity per channel is constant prior to clogging and does not responds to possible clogging events in neighboring channels^27^.

At this pressure difference the upper bound of the fluid Reynolds number becomes *Re*_*f*_ ~ 0.4, with a particle Reynolds number *Re*_*p*_ ~ 6 ⋅ 10^−3^ and a Peclet number *Pe* ~ 5 ⋅ 10^5^. These values where computed at the centre position of the bottleneck, where the flow velocity is maximum; they thus represent the upper bounds for these dimensionless numbers. This ensures complete laminar flow and domination of advective displacements of the particles^28^.

The flow through the channels is imaged with brighfield microscopy (Zeiss Axiovert 200) aqcuiring images at one frame per second. Examples of the images we obtain during the experiments can be found in Fig. 2a,b. To extract quantitative data from the movies we process the images with custom analysis routines, which are available upon request. The first step in the analysis is the construction of a kymograph, in which one horizontal row of pixels is plotted as a function of time for consecutive frames. From these we can automatically recognise when and where a clogging event occurs as this leads to a distinct change in the light transmission both upstream and downstream from a clog. We also determined the local flow fields using Particle Imaging Velocimetry (PIV) by recording a high-speed (2300 fps) movie around a single constriction with a high-speed camera (Phantom v9.1).

## Additional Information

**How to cite this article**: van de Laar, T. *et al*. Transition-state theory predicts clogging at the microscale. *Sci. Rep.* **6**, 28450; doi: 10.1038/srep28450 (2016).

## References

[b1] de Boer R. B., Leerlooyer K., Eigner M. R. P. & van Bergen A. R. D. Screening of Crude Oils for Asphalt Precipitation: Theory, Practice, and the Selection of Inhibitors. Society of Petroleum Engineers 10, 55–61 (1995).

[b2] Chang I.-S., Clech P. L., Jefferson B. & Judd S. Membrane fouling in membrane bioreactors for wastewater treatment. Journal of Environmental Engineering 128, 1018–1029 (2002).

[b3] Ang W. S. & Elimelech M. Protein (BSA) fouling of reverse osmosis membranes: Implications for wastewater reclamation. Journal of Membrane Science 296, 83–92 (2007). 10.1016/j.watres.2008.07.032 18760439

[b4] Griffiths I. M., Kumar A. & Stewart P. S. A combined network model for membrane fouling. Journal of colloid and interface science 432, 10–8 (2014). 25042380 10.1016/j.jcis.2014.06.021

[b5] Genovese D. & Sprakel J. Crystallization and intermittent dynamics in constricted microfluidic flows of dense suspensions. Soft Matter 7, 3889–3896 (2011).

[b6] Wyss H. M., Blair D. L., Morris J. F., Stone H. A. & Weitz D. A. Mechanism for clogging of microchannels. Phys. Rev. E 74, 61402 (2006). 10.1103/PhysRevE.74.061402 17280068

[b7] Linkhorst J., Beckmann T., Go D., Kuehne A. J. C. & Wessling M. Microfluidic colloid filtration. Scientific Reports 6, 22376 (2016). 26927706 10.1038/srep22376 PMC4772133

[b8] Baxter G. W. & Behringer R. P. Cellular automata models of granular flow. Physical Review A 42, 1017 (1990). 10.1103/physreva.42.1017 9904123

[b9] Nagel K. & Paczuski M. Emergent traffic jams. Phys. Rev. E 51, 2909–2918 (1995). 10.1103/physreve.51.2909 9962967

[b10] Helbing D. Traffic and related self-driven many-particle systems. Rev. Mod. Phys. 73, 1067–1141 (2001).

[b11] Schadschneider A. . Evacuation dynamics: Empirical results, modeling and applications. In Encyclopedia of complexity and systems science , 3142–3176 (Springer, 2009).

[b12] Pastor J. M. . Experimental proof of faster-is-slower in systems of frictional particles flowing through constrictions. Phys. Rev. E 92, 62817 (2015). 10.1103/PhysRevE.92.062817 26764754

[b13] Zuriguel I. . Clogging transition of many-particle systems flowing through bottlenecks. Scientific Reports 4, 7324 (2014). 25471601 10.1038/srep07324 PMC4255180

[b14] Lim A. Membrane fouling and cleaning in microfiltration of activated sludge wastewater. Journal of Membrane Science 216, 279–290 (2003).

[b15] Mukhopadhyay R. When microfluidic devices go bad. Analytical chemistry 77, 429—-A (2005). 16285143

[b16] Sauret A. . Clogging by sieving in microchannels: Application to the detection of contaminants in colloidal suspensions. Applied Physics Letters 105 (2014).

[b17] Bacchin P., Marty A., Duru P., Meireles M. & Aimar P. Colloidal surface interactions and membrane fouling: Investigations at pore scale. Advances in Colloid and Interface Science 164, 2–11 (2011). 21130419 10.1016/j.cis.2010.10.005

[b18] Rinne H. The Weibull distribution: a handbook (CRC Press, 2008).

[b19] To K., Lai P.-Y. & Pak H. K. Jamming of Granular Flow in a Two-Dimensional Hopper. Phys. Rev. Lett. 86, 71–74 (2001). 11136096 10.1103/PhysRevLett.86.71

[b20] Dinther A. M. C., Schroën C. G. P. H., Imhof A., Vollebregt H. M. & Boom R. M. Flow-induced particle migration in microchannels for improved microfiltration processes. Microfluidics and Nanofluidics 15, 451–465 (2013).

[b21] Hänggi P., Talkner P. & Borkovec M. Reaction-rate theory: fifty years after Kramers. Rev. Mod. Phys. 62, 251–341 (1990).

[b22] Kramers H. A. Brownian motion in a field of force and the diffusion model of chemical reactions. Physica 7, 284–304 (1940).

[b23] Paine A. J., Luymes W. & McNulty J. Dispersion polymerization of styrene in polar solvents. 6. Influence of reaction parameters on particle size and molecular weight in poly(N-vinylpyrrolidone)-stabilized reactions. Macromolecules 23, 3104–3109 (1990).

[b24] Lekkerkerker H. N. W. & Tuinier R. Colloids and the depletion interaction , vol. 833 (Springer, 2011).

[b25] Yodh A. G. . Entropically driven self-assembly and interaction in suspension. Philosophical Transactions of the Royal Society of London A: Mathematical, Physical and Engineering Sciences 359, 921–937 (2001).

[b26] Xia Y. & Whitesides G. M. Soft Lithography. Angewandte Chemie International Edition 37, 550–575 (1998). 10.1002/(SICI)1521-3773(19980316)37:5<550::AID-ANIE550>3.0.CO;2-G 29711088

[b27] Acheson D. J. Elementary fluid dynamics (Oxford University Press, 1990).

[b28] Squires T. M. & Quake S. R. Microfluidics: Fluid physics at the nanoliter scale. Rev. Mod. Phys. 77, 977–1026 (2005).

